# Issues of AI and human resource development: applications in education and the arts

**DOI:** 10.3389/frai.2025.1619980

**Published:** 2025-10-17

**Authors:** Eun Jin Jung, Hea Jun Yoon

**Affiliations:** Korea Research Institute for Vocational Education and Training, Sejong, Republic of Korea

**Keywords:** artificial intelligence, human resource development, creativity, AI-human synergistic creativity and learning framework, ethics

## Abstract

This study examines how artificial intelligence (AI) transforms human learning, creativity, and ethical engagement, with implications for future Human Resource Development (HRD). Drawing on Computational Creativity theory, a cross-domain case study analysis—including educational tools (e.g., Jill Watson, Cognii) and generative art platforms (e.g., AICAN, DALL·E)—reveals the dual role of AI as both cognitive collaborator and autonomous agent. The paper structures its discussion around three key dimensions: education (personalized learning vs. development of metacognitive competence), arts (co-authorship dilemmas vs. preservation of human originality), and ethics (regulatory gaps in professional education). Through these domains, the study highlights interdependent tensions and synergies, and argues that AI integration calls for reconceptualizing human–machine interaction in HRD. It then proposes the AI–Human Synergistic Creativity and Learning Framework, which emphasizes collaborative creativity, ethical reflection, and adaptive learning for workforce development. The findings offer actionable insights into curriculum design, policy formulation, and institutional training strategies in AI-augmented contexts.

## 1 Introduction

Artificial Intelligence (AI) is defined as a computing system capable of performing functions such as learning, adaptation, synthesis, self-correction, and data utilization for complex processing tasks—functions that resemble human cognitive processes (Popenici and Kerr, 2017). Machine learning refers to the study of computer algorithms that improve automatically through experience. It is a technology that analyzes vast amounts of data to predict future outcomes and is considered a subfield of artificial intelligence. Through deep learning, AI has reached a level where it can learn autonomously by utilizing large-scale data (Aggarwal, 2018). Algorithms, machine learning, and artificial neural networks are known as the three key factors of AI (Abu Owda et al., 2023).

Generative Artificial Intelligence (GAI) extends beyond traditional analytical AI—which is primarily concerned with data analysis and pattern recognition—by enabling the creation of entirely new content. GAI refers to AI technologies capable of generating novel outputs based on existing information. It is widely used in various programs such as computer graphics and natural language processing, leading to significant transformations in these fields. ChatGPT, Bard (Gemini)—a conversational generative AI chatbot developed by Google to directly compete with OpenAI’s ChatGPT—LLAMA, a large-scale language model released by Meta AI in February 2023, and Claude, a large-scale language model developed by Anthropic (a U.S.-based AI startup), are all categorized as Large Language Models (LLMs). These models are artificial neural network-based language models with a vast number of parameters that learn values from data. They are trained on large amounts of unlabeled text and use prompt-based textual imputation to statistically predict the sequence of words based on a given input, providing responses accordingly.

In essence, AI has been anticipated to discover new solutions to given problems and offer novel perspectives. This perspective has driven the advancement of the field known as Computational Creativity (CC), which involves modeling or simulating creativity using computers. As interest in AI continues to grow, both academia and industry are closely examining how much of human capability AI can eventually replace.

The advancement of AI technology has had a profound impact across various fields, with education and the arts playing particularly significant roles. In the field of education, AI is introducing new methods such as personalized learning, efficient educational management, and improved accessibility, fundamentally transforming students’ learning experiences. In the arts, AI serves as a creative tool, providing artists with new sources of inspiration and enabling the creation of innovative works that challenge traditional artistic forms. For instance, in 2018, when the AI-generated portrait Edmond de Belamy was sold at a Christie’s auction for approximately $432,000, it sparked debates over the ownership of AI-created artworks.

These two fields—education and the arts—serve as critical domains that illustrate AI’s multifaceted impact on both cognitive and creative processes. While prior research has examined AI applications in these areas, most studies remain confined within disciplinary silos and tend to focus narrowly on either technological functionality or ethical considerations (Zawacki-Richter et al., 2019). There is a lack of integrative perspectives that explore how AI simultaneously mediates learning and creativity across domains, and limited theorization on its implications for Human Resource Development (HRD), particularly in redefining these processes as collaborative endeavors between humans and machines.

To address these gaps, this study adopts a cross-domain analytical framework that examines how AI reshapes educational and artistic practices. Through selected case studies—Thinkster Math, Cognii, and Jill Watson in education; GAN, DeepDream, AICAN, and DALL·E in the arts—the research identifies emerging HRD challenges and proposes a conceptual model that frames AI as a co-agent in creativity and learning. The study ultimately offers practical insights for designing future HRD strategies suited for AI-integrated environments.

## 2 Methods and materials

To examine how AI reshapes educational and artistic practices, this study employed a qualitative case study approach using purposive sampling. The selected cases meet three criteria: (1) representativeness, (2) diversity across domains, and (3) documented impact. For the education domain, Thinkster Math, Cognii, and Jill Watson were chosen as they represent distinct pedagogical approaches—namely, AI-driven tutoring, formative feedback, and instructional support. In the arts, GAN, DeepDream, Neural Style Transfer, AICAN, and DALL·E were selected to reflect a wide spectrum of generative, interpretive, and collaborative creativity mediated by AI (Cetinic and She, 2022; Mineo, 2023).

Thinkster Math is an AI-powered tutoring platform that delivers personalized mathematics instruction for students from kindergarten through high school. By analyzing individual learning patterns, it generates tailored lesson plans to strengthen each student’s understanding. Initially launched as Tabtor Math in 2010 in New Jersey, it has since expanded to over 30 countries, serving more than 70,000 students. Cognii provides AI-based learning solutions for K–12 education, higher education, and corporate training. Its virtual learning assistants offer immediate, adaptive feedback, helping learners to refine their conceptual understanding. Founded in 2013 and based in San Francisco, Cognii emphasizes personalized, interactive learning experiences. Jill Watson is a virtual teaching assistant developed in 2016 at the Georgia Institute of Technology. Built on IBM’s Watson platform, it uses natural language processing to automatically respond to student inquiries in online learning environments, thereby automating certain aspects of instruction and learner support.

In the domain of art, Generative Adversarial Networks (GANs), introduced in 2014 by Ian Goodfellow, enable machines to create new visual content through adversarial learning mechanisms. DeepDream, developed by Google in 2015, transforms ordinary images into dream-like, surreal artworks using deep convolutional networks. Neural Style Transfer applies the style of a given artist to other visual content, allowing users to produce painterly reinterpretations. AICAN, created by researchers at Rutgers University in collaboration with Facebook, learns from large art datasets to generate novel artworks that blend multiple stylistic influences. Finally, DALL·E, developed by OpenAI, generates original images from text prompts, combining language comprehension with visual creativity.

For each case, document analysis was conducted as the primary method of inquiry, drawing on peer-reviewed articles, official websites and media coverage. The analysis focused on three dimensions: (1) the core AI technology utilized, (2) the mode of human-AI interaction, and (3) the resulting pedagogical or creative outcomes. This approach follows Yin (2018) guidelines for explanatory case study research and enables cross-case synthesis to derive broader implications for Human Resource Development (HRD) in AI-integrated contexts.

## 3 Results

### 3.1 Case study on the educational applications and transformations of AI

#### 3.1.1 Thinkster Math

Thinkster Math (Thinkster, 2025) is a personalized math tutoring program that combines human interaction with AI to provide customized learning experiences. The AI utilized in this program collects data related to factors such as the time students take to solve problems and the accuracy of their answers, helping teachers develop the next stages of learning content. AI and machine learning—computer algorithms that automatically improve through experience—are used to analyze vast amounts of data and predict future outcomes, making them a key area of artificial intelligence. The combination of data-driven insights powered by AI and Thinkster Math’s patented ART (Active Replay Technology) creates a highly effective learning system. With this technology, teachers can create personalized learning plans tailored to each student’s needs. Thanks to these advantages, Thinkster Math is currently used by tens of thousands of students across more than 40 countries (Thinkster, 2025).

Thinkster Math’s teaching methodology can be summarized in seven key steps: teaching math in a way that ensures lifelong retention, creating fully personalized content for each student, providing elite teachers for daily monitoring, coaching, and feedback, delivering insights into student performance to enhance mastery and accelerate learning, offering timely assistance in problem-solving, ensuring continuous student engagement, and making math learning a comfortable experience. In addition to these seven steps, Thinkster Math rigorously integrates three core elements: expert math teachers, advanced technology, and personalized learning plans.

The advantages of Thinkster Math include: (1) establishing personalized learning plans for each student to bridge the technology gap, (2) providing one-on-one math tutoring with certified math coaches, (3) incorporating gamified problems across various subtopics, (4) enabling students to develop smart learning techniques and positive behavioral changes, (5) utilizing patented AI technology to guide learning and problem-solving, allowing concept acquisition in just 15 min a day, and (6) enabling ultra-fast learning through real-time performance tracking and automated grading.

#### 3.1.2 Cognii

Cognii provides a virtual learning assistant platform that engages in real-time conversations with students. As a leading provider of AI-based educational technology, Cognii collaborates with organizations in the higher education and corporate training sectors to deliver superior learning outcomes. It plays a significant role in enhancing global education by supporting personalized deep learning, intelligent tutoring, open-ended response assessment, and comprehensive analytics. Cognii’s Virtual Learning Assistant (VLA) prompts students to provide answers and offers instant feedback on their responses (Cognii, 2025). Additionally, it provides personalized hints to encourage student engagement through chatbot-style interactions. VLA can be used independently or in combination with the Cognii Analytics program, which summarizes students’ comprehension levels and interactions with VLA to provide valuable insights to educators.

Cognii not only provides feedback for learning but also plays a crucial role in learning assessment. Its ability to process deeper natural language allows it to interpret students’ lengthy responses using semantic variations. Additionally, by leveraging data mining and machine learning, Cognii continuously improves the accuracy of its grading and feedback over time. In contrast, virtual assistants like Siri and Alexa primarily handle shallow natural language, as they process relatively short user inputs.

The key features and advantages of Cognii can be summarized in approximately seven points. First, it promotes “open response.” Rather than responding to multiple-choice questions, students articulate their thoughts and answers in their own words and expressions when engaging with open-ended questions. This approach allows students to build their unique knowledge base while also fostering critical thinking skills.

Second, ‘formative assessment’ is possible. Formative assessment is conducted with the goal of improvement, focusing on the learning process rather than just the final results or performance. Through Cognii, students receive real-time feedback throughout their learning journey, while teachers gain in-depth insights into individual student achievement levels and problem-solving processes.

Third, “one-on-one tutoring” is possible. Students can engage in extended conversations with Cognii and receive various forms of coaching. Cognii allows students to make multiple attempts until they master a concept, ultimately supporting the Bloom’s 2-sigma phenomenon. Bloom’s 2-sigma, identified by Bloom (1984), refers to the finding that students who receive one-on-one tutoring or individualized instruction achieve learning outcomes comparable to the top 2% of students in traditional classroom lectures—meaning that one-on-one instruction is, on average, two standard deviations more effective than conventional classroom teaching.

Fourth, “personalization” becomes possible. Since Cognii conducts open-response assessments with high accuracy, it can provide “personalized learning pathways” tailored to each student’s level. Based on this, it also offers content-specific, one-on-one coaching to meet individual student needs.

Fifth, “scalability” is a significant advantage. Since Cognii supplements the roles of teachers, instructors, and graders, it enables the large-scale delivery of high-quality education. Ultimately, by making education more accessible, it helps lower barriers to learning for students. From this perspective, the scalability of Cognii is remarkably high.

Sixth, Cognii enhances student retention and course completion rates. By actively promoting student engagement and persistence, it significantly increases the likelihood that students will stay committed to and complete their courses. With personalized feedback and appropriately leveled follow-up questions, Cognii helps maintain students’ interest and motivation throughout the learning process.

Seventh, Cognii also assesses students’ critical thinking skills. The open-ended questions encourage students to think independently and articulate their responses. Through this process, students engage in higher-order thinking to formulate their answers. Cognii evaluates students’ critical thinking based on their responses and provides appropriate feedback accordingly.

As described above, the benefits that students gain from Cognii are diverse. Through Cognii, students enhance their learning effectiveness, engagement, and immersion, while teachers can reduce the time and energy spent on routine tasks. Additionally, with instant grading and feedback, teachers can provide personalized coaching tailored to each student’s needs. The technology used in Cognii differs from other essay-scoring software. Most conventional programs only assign simple scores or evaluate writing structure and style without offering detailed feedback. In contrast, Cognii engages students in idea-driven conversations, encouraging participation and allowing them to receive real-time feedback, making multiple attempts until they achieve mastery.

Cognii is also known to be applicable in higher education institutions (universities). It can be used to design and develop AI-based online courses and serve as an assessment tool to enhance teaching efficiency. Additionally, it can be integrated into real-time tutoring platforms that students can access anytime and anywhere, as well as systems for evaluating student writing. Cognii enables effective practice and assessment to improve students’ problem-solving and communication skills while also providing analytics to identify their needs. Furthermore, it offers students a unique and engaging learning experience.

Based on the diverse applications of Cognii, universities will be able to develop methods for providing high-quality education to a large number of students, while students can engage in highly immersive learning experiences tailored to their educational needs. At-risk students can also be identified, allowing for the provision of individualized support at appropriate levels. In this regard, Cognii is expected to have a positive impact on the higher education market as well.

#### 3.1.3 Virtual teaching assistant: Jill Watson

Jill Watson is a virtual teaching assistant (VTA) powered by AI, introduced by the Georgia Institute of Technology in 2016. In the summer of 2015, it was developed to support the Online Master of Science in Computer Science (OMSCS) program at Georgia Tech and assist with online courses related to knowledge-based artificial intelligence within OMSCS. Jill Watson was created by Professor Ashok Goel and a team of graduate students from the Design and Intelligence Laboratory (DILAB) at Georgia Tech.

In the spring of 2016, Jill Watson joined the teaching staff of the Knowledge-Based Artificial Intelligence—Cognitive Systems (KBAI) course. In the online forum, Jill Watson diligently answered numerous student questions with a high level of accuracy. As a result, students did not realize that Jill Watson was an AI.

**Figure 1:**
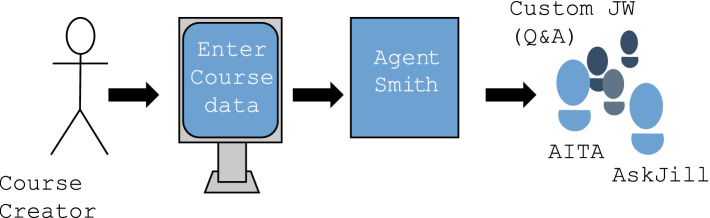
Jill Watson’s role as Agent Smith. Goel (2020, p. 3).

Examining the evolution of Jill Watson, the initial creation time was 1,500 h, whereas by the spring of 2020, it had been reduced to just five hours. Since 2019, the introduction of Agent Smith has enhanced its role as an interactive knowledge-based expert. Agent Smith is an interactive, knowledge-based expert that facilitates the rapid training of domain-specific Q&A systems. By efficiently adapting to new domains, it helps save hundreds of hours and is responsible for generating knowledge bases from documents such as new course syllabi (see Figure 1). As a result, the number of courses in which Jill Watson served as a teaching assistant increased to four in the fall of 2019, and by that year, its application expanded to the field of biotechnology.

Jill Watson is capable of expanding its own data through self-learning based on machine learning. Its ability to provide instant responses to students’ various questions without time or environmental constraints, utilizing advanced natural language processing, data analysis, and machine learning-based data expansion, is highly innovative. Initially, students found Jill Watson’s responses somewhat unnatural. However, after rapid learning, it began providing answers with approximately 97% accuracy. Thanks to its advancements, students came to prefer asking Jill Watson questions, as its responses became not only highly accurate but also instantaneous and available 24/7. This led to a positive effect, increasing student engagement and participation in class through interactions with Jill Watson.

Currently, professor Ashok Goel’s team and Georgia Tech are preparing to expand the AI teaching assistant system to various departments. In the School of Biological Sciences at Georgia Tech, they have developed VERA (Virtual Ecological Research Assistant), based on Jill Watson, to support graduate-level research. VERA is an application that allows users to construct conceptual models of ecological systems and run interactive simulations based on those models. This enables researchers to explore ecosystems, conduct virtual experiments, and predict future changes and outcomes (VERA Beta, 2024).

**Figure 2:**
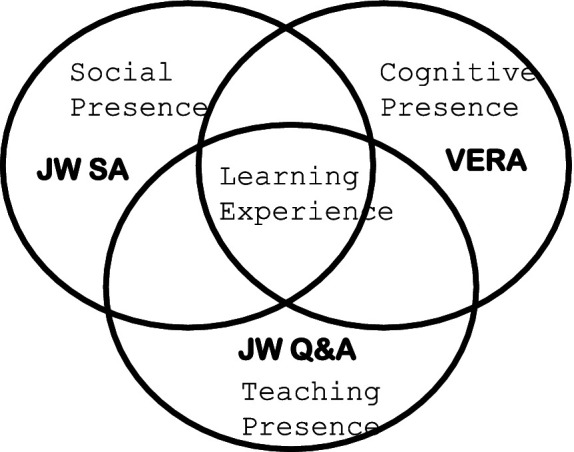
Three factors of Jill Watson. Goel (2020, p. 13).

Ultimately, one of VERA’s main goals is to create an accessible tool for scientific inquiry. Currently, research funding for the VERA project is supported by a grant from the National Science Foundation (NSF). The provided (Figure 2) categorizes the learning experiences pursued through Jill Watson into three factors. The first is Jill Watson SA (Social Assistant), which plays a role in enhancing social presence. In practice, it serves as a virtual social assistant that connects learners in the OMSCS (Online Master of Science in Computer Science) program. Jill Watson SA also helps form small communities alongside Jill Watson Q&A.

The second factor is Jill Watson Q&A, which enhances the presence of instruction. Acting as a virtual teaching assistant, it automatically responds to various questions arising in OMSCS courses and even serves a role similar to that of a teacher in online classes. The third factor is VERA (Virtual Ecological Research Assistant), which is responsible for cognitive presence. As a virtual research assistant, it supports online experiments by utilizing a biological encyclopedia and enhances cognitive presence by leveraging open-source educational materials available online.

These three factors—social presence, cognitive presence, and teaching presence—also represent the direction in which AI technology has evolved in online education. In fact, Garrison et al. (2010) emphasized the need to enhance social, cognitive, and teaching presence in online learning environments.

The pedagogical implications of Jill Watson can be further understood through the lens of the Community of Inquiry framework, as described below. The Jill Watson case also aligns closely with the Community of Inquiry (CoI) framework, which conceptualizes effective online learning through the interplay of three core elements: cognitive presence, teaching presence, and social presence (Garrison et al., 1999). Jill Watson, as a virtual teaching assistant, contributes significantly to all three components. It enhances cognitive presence by facilitating knowledge construction through Q&A interactions; it supports teaching presence by delivering consistent, context-aware responses that simulate instructor guidance; and it fosters social presence through its 24/7 availability and engagement with learners in discussion forums. This connection between Jill Watson and the CoI framework illustrates how AI can be meaningfully embedded in pedagogical models traditionally designed for human-mediated learning, thus opening new directions for both instructional design and AI integration in education.

### 3.2 Case analysis of AI and art

AI is bringing significant changes to the way art is created. Artists are utilizing AI tools to develop new forms of art, and many are captivated by AI’s capabilities, actively seeking ways to collaborate with it. AI offers artists a wide range of possibilities, such as generating complex patterns and designs through algorithms or analyzing existing works to create new styles. With these functionalities, artists can reduce repetitive tasks and focus more on the creative aspects of their work.

**Figure 3:**
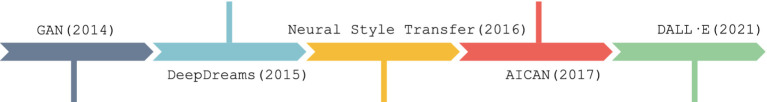
A significant technological milestone that led to the emergence of contemporary AI-generated art. Cetinic and She (2022, p. 6).

Recently, at a Christie’s art auction—one of the world’s most renowned auction houses, established in 1766—a portrait generated by artificial intelligence was sold for approximately $432,000, which is around 500 million KRW. Initially, people expected it to be priced at around $10,000, making the final bid nearly 50 times higher than anticipated. This has raised questions about how credit and responsibility should be assigned and how AI systems have successfully created artwork. Furthermore, the advancement and application of AI in the field of art bring ethical concerns, particularly regarding copyright issues and the identity of creators. Efforts to address these challenges are now more necessary than ever. Before delving into these discussions, it is essential to first examine the technological milestones that have led to the modern production of AI-generated art (see Figure 3).

#### 3.2.1 GAN: generative adversarial network

The Generative Adversarial Network (GAN) is a machine learning model introduced in 2014 by Ian Goodfellow and his colleagues. GANs have achieved significant success in the field of image generation by utilizing two neural networks. The two key components of a GAN are the Generator and the Discriminator. The Generator takes a random noise vector as input and generates images, with the ultimate goal of creating images that are indistinguishable from real data. The Discriminator, on the other hand, receives both real data and generated data as input and determines which is real and which is fake, playing a crucial role in improving the Generator’s performance.

These two neural networks improve their performance through competition: the Generator strives to create more realistic images to deceive the Discriminator, while the Discriminator continuously learns to better distinguish between real and fake images. Based on this mechanism, GANs have brought innovation to various fields, including image generation, artistic creation, and data augmentation. Artists experiment with GANs to create new forms of art, while the film industry utilizes them to enhance special effects and animation.

#### 3.2.2 DeepDream

DeepDream, an algorithm developed by Google’s research team in 2015, is known for creating unique artistic effects by repeatedly enhancing features within an image. It identifies patterns in an image and exaggerates them, generating dream-like, surreal visuals that resemble hallucinatory experiences.

DeepDream utilizes a Convolutional Neural Network (CNN) to process images, detecting various features as they pass through multiple layers of the network. Additionally, it serves as a data visualization tool, helping to visually represent and interpret complex structures or patterns.

#### 3.2.3 Neural style transfer

Neural style transfer is a technique that utilizes deep learning to apply one artistic style to another image. This allows users to transform their photos or images into the style of a specific artist. The concept was introduced by Leon A. Gatys in 2015. Deep learning-based style transfer has served as a starting point for creative applications in computer vision.

Style transfer operates by combining two loss functions: content loss and style loss. The content loss function measures how well the generated image preserves the original content, while the style loss function evaluates how accurately the generated image replicates the artistic style. For example, if a nighttime view of the Han River is used as the content image and Vincent van Gogh’s Starry Night is used as the style image, the result would be a transformed Han River nightscape with Van Gogh’s distinctive brushstrokes and color palette. This transformation is achieved through neural style transfer.

#### 3.2.4 AICAN

AICAN (Artificial Intelligence Creative Adversarial Network) is an AI-based creative system developed by researchers at Rutgers University in New Jersey and Facebook. The development team created the Creative Adversarial Network (CAN), an advanced form of the Generative Adversarial Network (GAN), and integrated AI technology to complete AICAN. Unlike GANs, AICAN possesses creativity. While it learns from existing artists’ works, it strives to create entirely new and unique pieces. In fact, about 75% of viewers found it difficult to distinguish AICAN’s artworks from those created by human painters, demonstrating its impressive artistic quality and creativity (Smart Content Center, 2021). As an AI capable of generating artworks, AICAN ultimately focuses on fostering collaboration between humans and AI in the creative process.

One of the key features of AICAN is that it is based on the Generative Adversarial Network (GAN). As previously mentioned, GAN consists of two neural networks: a generator that creates images and a discriminator that determines whether the generated images are real or fake. These two networks compete against each other, continuously improving their performance.

AICAN is highly proficient at learning various artistic styles and techniques by training on a large dataset of artworks. When generating new pieces, it can either mimic existing artistic styles or create entirely new ones. Additionally, it introduces creative variations to produce original works, similar to how human artists explore new ideas and artistic styles.

#### 3.2.5 DALL-E

DALL-E is an image-generating AI model developed by OpenAI in 2021. The name “DALL-E” is a combination of “Dali” and “WALL-E,” inspired by the surrealist painter Salvador Dalí and the animated movie character WALL-E from Pixar. This fusion symbolizes DALL-E’s technological integration and creativity. DALL-E generates images based on text descriptions provided by users. Built on a vast language model with billions of parameters, it can comprehend diverse textual inputs and create corresponding images. Depending on user input, it can render images in various styles, such as cartoons, photographs, and surrealism.

The applications of DALL-E are highly diverse. In addition to assisting in the creation of unique visual content in the fields of design and art, it can also be utilized for educational and research purposes. It enables the visualization of complex concepts and the illustration of new research ideas. Furthermore, DALL-E can be used in advertising and marketing by generating original and captivating content, which helps enhance brand image.

These examples of AI-generated art can be further understood through the lens of creativity theory, particularly Rhodes’ 4P model. The application of AI in the arts can also be interpreted through Rhodes’ 4P model of creativity, which conceptualizes creativity as the interaction of Person, Process, Product, and Press (Rhodes, 1961). In this context, AI systems such as DALL·E or AICAN do not merely replace human creators, but instead interact with each of the four components. For instance, while the Person traditionally referred to the individual artist, AI now functions as a co-creator or creative extension of the artist. The Process of creation is transformed through algorithmic generation and iteration. The Product becomes more fluid and data-driven, often challenging traditional definitions of authorship. Lastly, the Press, or environmental and cultural context, is reshaped by public discourse around AI-generated art, intellectual property, and aesthetic legitimacy. By mapping AI-enabled art practices to the 4P model, this study emphasizes that creativity in the AI era is not solely about human originality but emerges from new forms of interaction between humans, machines, and sociocultural environments.

## 4 General discussion

Based on the previously mentioned case studies, it is crucial to identify and summarize the competencies that humans need to cultivate as AI becomes increasingly integrated into education and the arts. This study aims to highlight critical thinking, creativity, and ethics as essential competencies in this context. In addition to identifying these core competencies, this study also contributes to the field of Human Learning and Behavior Change by demonstrating how AI technologies, particularly generative AI, can be designed and integrated to enhance core mechanisms of learning such as metacognitive awareness, intrinsic motivation, and critical thinking. By analyzing educational and creative AI applications, the study reveals how AI can act not merely as a content provider but as a co-agent that stimulates higher-order cognitive engagement and sustained behavioral change.

Recent literature emphasizes that learner–AI interaction should be viewed not merely as a technical process but as a psychological and behavioral experience that influences learners’ reflection, persistence, and growth (Zeb et al., 2024; AVID Open Access, 2025a). These insights inform the design of AI-integrated interventions aimed at fostering active learning and long-term behavioral change. Generative AI tools such as ChatGPT not only facilitate knowledge dissemination but also promote ethical engagement and academic reflection (Zeb et al., 2025). Moreover, integrating high-performance HR practices within supportive organizational cultures is essential for innovation, particularly in AI-driven learning and development environments (Zeb et al., 2022). Finally, ethical and just organizational contexts significantly enhance job performance through high-performance HR practices, reinforcing the need for trust and fairness in AI-integrated HRD strategies (Zeb et al., 2021).

Building on these behavioral insights, this study further deepens its theoretical framing by interpreting the selected cases through the lens of Computational Creativity (CC). CC posits that creativity—traditionally considered a uniquely human trait—can be modeled, simulated, or co-generated by machines (Colton and Wiggins, 2012). In this study, education-focused tools such as Jill Watson and Cognii reflect ‘supportive creativity,’ where AI augments human thinking through responsive feedback and intelligent questioning. In contrast, AI art generators like AICAN and DALL·E embody aspects of ‘autonomous creativity,’ as they independently generate novel outputs with aesthetic or conceptual value. By applying CC theory across domains, the study highlights how AI not only performs creative functions but also challenges the boundaries of what constitutes creativity in learning and professional contexts.

This theoretical framing also offers implications for Human Resource Development (HRD). As AI systems take on creative and cognitive roles, fostering human-AI collaborative creativity becomes a central task of future HRD strategies. Rather than positioning creativity as a purely innate or human-exclusive skill, CC suggests that creativity can be distributed across systems—opening new possibilities for how organizations train for innovation, critical inquiry, and design thinking.

To translate these theoretical insights into practical implications, the study examines three critical dimensions—cognitive development, creative capacity, and ethical awareness—through which AI-integrated learning environments can be reimagined. Each dimension reflects a unique facet of human-AI interaction and suggests targeted strategies for future Human Resource Development (HRD).

First, Cognitive competence encompasses critical thinking, analytical reasoning, and problem-solving abilities. This study focuses on critical thinking, particularly as it pertains to interacting with generative AI. In the AI era, the ability to critically evaluate information has become increasingly vital to ensure AI is used responsibly and effectively (Carucci, 2024).

To cultivate cognitive competence, students must learn to formulate precise prompts by identifying key information and terms that elicit relevant AI responses. They should also be able to assess the quality of their prompts and critically evaluate AI-generated outputs for logic, accuracy, and bias, complementing them with independent research. This iterative refinement process fosters self-regulated learning and deeper cognitive engagement. Furthermore, students need to develop a critical understanding of AI’s capabilities and limitations. When direct AI use is constrained, educators can support engagement through age-appropriate discussions and reflective activities that promote metacognition. Through these strategies, students not only strengthen their critical thinking but also learn to navigate AI tools more effectively. This iterative interaction fosters deeper learning and promotes a sustainable cycle of cognitive development. Accordingly, schools should support this process through structured curricula and pedagogical guidelines that integrate AI meaningfully into education (AVID Open Access, 2025b).

Second, although general competencies encompass creativity, communication, and collaboration, this paper places particular emphasis on creativity—the capacity to generate novel ideas, concepts, and solutions (Amabile and Pratt, 2016). In the AI era, human creativity is increasingly recognized as a critical and irreplaceable capability.

Fostering creativity in education requires attention to several pedagogical elements. First, creative tasks naturally enhance motivation and engagement, especially when integrated with AI tools. Second, cognitively stimulating activities promote higher-order thinking and support creative problem-solving. Third, creative processes contribute to emotional development by fostering resilience, empathy, and interpersonal skills through collaboration. Fourth, inclusive strategies should ensure that students facing barriers to AI access also benefit from creative opportunities. Finally, creativity must be linked to future job competencies, as original thinking remains a uniquely human asset amid increasing automation. Therefore, creativity should be emphasized not only as a cognitive skill but also as a core component of career readiness. This necessitates curriculum innovation and long-term support for creative thinking as a foundation for lifelong learning (AVID Open Access, 2025c).

Third, the ethical implications of AI remain a critical area of discourse. While universal ethical principles for AI have yet to be established, collaborative efforts across academia, government, and industry continue to shape foundational guidelines. Key principles emphasize the protection of human dignity, human oversight, transparency, data privacy, and inclusivity.

Ethical concerns surrounding AI-generated art are increasingly prominent, particularly regarding intellectual ownership. Since AI models like GANs rely on existing data, questions arise over who holds copyright—the developer, data provider, or user. The 2018 Christie’s auction of *Edmond de Belamy* exemplified such debates, as have projects like Google’s *Magenta* and Sony’s *Flow Machines*, where legal rights were ultimately assigned to the human creators behind the AI. Another key issue involves whether AI-generated outputs constitute “true” art. As AI replicates creative processes, the artist’s role and identity are being reexamined. Nonetheless, the irreplaceable nature of human creativity highlights the importance of using AI to augment rather than replace artistic expression. Recent interviews with artists suggest a shift in perception—from resistance to collaboration. Some liken AI to a collective unconscious (Jung), capable of offering insights drawn from vast datasets beyond individual experience. Others emphasize the importance of openness toward AI, viewing it as a human-made tool that, if approached creatively, can expand artistic possibilities. Such perspectives highlight a growing recognition of AI as a collaborative partner in the creative process, rather than a threat (SAP, 2024).

Building upon these foundational competencies, the following section examines how such challenges and opportunities are manifesting across key domains—education, the arts, and professional ethics—each of which poses distinct implications for future HRD strategies.

### 4.1 Education: personalized learning vs. development of metacognitive competence

One of the most significant contributions of AI in education is its capacity to personalize learning by adapting to individual learners’ needs. Intelligent tutoring systems such as Thinkster Math and Cognii exemplify how AI can diagnose learners’ strengths and weaknesses, deliver customized content, and provide real-time feedback, thereby enhancing engagement and learning efficiency. However, while personalization improves short-term outcomes, it may inadvertently hinder the development of metacognitive competence—the ability of learners to reflect on, monitor, and regulate their own cognitive processes. Such skills are essential for deep and sustained learning, particularly in AI-mediated environments where individuals must critically evaluate the credibility, relevance, and reliability of AI-generated content. Therefore, future HRD strategies should not only utilize the adaptive strengths of AI, but also prioritize the design of learning environments that promote self-regulation, reflective inquiry, and critical thinking.

### 4.2 Arts: co-authorship dilemmas vs. preservation of human originality

In the creative arts, AI has emerged not merely as a tool but as a co-creator. Generative models such as GAN, AICAN, and DALL·E produce novel and aesthetically compelling works, thereby challenging traditional notions of authorship, originality, and artistic intent. The 2018 sale of *Edmond de Belamy*, an AI-generated portrait, exemplified these debates, raising questions about whether creative ownership should reside with the algorithm, the programmer, or the user. While such developments enable new forms of hybrid creativity, they also introduce complex legal and philosophical issues regarding co-authorship and intellectual property. Despite these challenges, the irreplaceable value of human originality—reflected in context-sensitive interpretation, emotional depth, and cultural nuance—remains central. Accordingly, future HRD strategies in the arts should preserve and nurture this human uniqueness while equipping individuals with the competencies necessary for collaborative creation with AI systems.

### 4.3 Ethics: regulatory gaps in professional education

Despite the growing integration of AI into educational and creative practices, ethical considerations are frequently addressed in fragmented or reactive ways. Key issues—including data privacy, algorithmic bias, plagiarism, and the misuse of AI-generated content—are rarely incorporated into educational curricula in a systematic manner. This highlights a broader regulatory gap in professional and higher education, where comprehensive guidelines for the responsible use of AI remain underdeveloped. As AI tools such as ChatGPT become increasingly prevalent in classrooms and workplaces, the lack of structured ethics education poses significant risks to both learners and institutions. To mitigate these risks, future HRD strategies must embed AI ethics education into formal training programs, incorporating principles of transparency, accountability, and fairness. Such integration is essential not only for promoting responsible AI adoption but also for preparing a workforce capable of navigating the complex moral and technical challenges of AI-augmented environments.

## 5 Conclusion

While much of the existing literature on AI focuses on its functional applications within isolated domains, this study adopts a cross-domain analytical framework to compare its roles in both education and the arts. This juxtaposition reveals how AI is not merely a supportive or efficiency-enhancing tool, but one that is increasingly embedded in creative and cognitive processes traditionally regarded as uniquely human. By doing so, the paper shifts the narrative from one of substitution to transformation—illustrating how AI challenges and reshapes the foundational assumptions of human-centered practices.

One of the study’s distinctive contributions lies in its conceptual reframing of Human Resource Development (HRD) in the context of AI integration. Rather than positioning AI as a competitor to human labor, the study explores how it can serve as a catalyst for enhancing human capacity. It highlights emerging competencies such as prompt engineering, meta-cognitive thinking, ethical literacy, and interdisciplinary adaptability as key to thriving in AI-augmented environments. These insights urge a departure from traditional skill-centered HRD models toward more dynamic, human-centered strategies that embrace collaboration with intelligent systems.

Additionally, this study provides practical implications by identifying new pedagogical and training approaches that align with the evolving human-AI interface. By analyzing case studies such as Thinkster Math, Cognii, and DALL·E, it suggests that future learning environments should not only integrate AI tools but also embed critical reflection and creativity as core learning objectives. These findings advocate for a transformative shift in how educators and institutions design curricula—moving beyond technical proficiency to fostering lifelong learning mindsets that are agile, ethically grounded, and future-ready.

Synthesizing these insights, this study proposes the “AI-Human Synergistic Creativity & Learning Framework,” which conceptualizes how AI systems—when embedded in learning and creative processes—act as cognitive and creative partners to human users. Drawing on Computational Creativity, the 4P model, and the Community of Inquiry framework, this model outlines how supportive and autonomous AI systems co-shape educational outcomes and creative production through dynamic human-AI interaction. The framework offers guidance for developing future HRD strategies that emphasize collaboration, ethical reasoning, and meta-cognitive skill development in AI-integrated environments.

### Future directions

This study explored how artificial intelligence (AI), particularly generative AI, is being integrated into the fields of education and the arts, and examined its implications for human learning, creativity, and human resource development (HRD). Through cross-case analysis of applications such as Thinkster Math, Cognii, Jill Watson, AICAN, and DALL·E, the research identified how AI functions not only as a technical tool but as an active agent that co-participates in cognitive and creative processes. These findings reveal that AI plays a dynamic role in facilitating individualized learning, enhancing critical thinking, and enabling new forms of artistic expression—often challenging traditional notions of authorship, expertise, and originality.

From a theoretical standpoint, this study contributes to the literature by linking real-world AI applications to key frameworks such as Computational Creativity, the Community of Inquiry model, and Rhodes’ 4P creativity model. By doing so, it presents a new conceptual lens to understand human-AI collaboration as a form of distributed creativity and learning. Practically, the study suggests that future HRD strategies must evolve beyond technical skill development and toward fostering collaborative, ethical, and meta-cognitive competencies in AI-integrated environments. The proposed conceptual framework—AI-Human Synergistic Creativity and Learning Framework—offers a starting point for institutions and educators seeking to reimagine workforce training and educational design in the age of AI.

Nevertheless, this study has certain limitations. It primarily relied on document-based qualitative case analysis, which, while useful for conceptual exploration, would benefit from empirical validation in future research. Follow-up studies could incorporate interviews, classroom-based experiments, or longitudinal analysis to better understand how learners and professionals engage with AI in real settings. Moreover, comparative studies across cultural or institutional contexts would deepen our understanding of how sociotechnical systems shape the role of AI in human learning and creativity. These future directions will help refine the theoretical and practical contributions outlined in this study and offer more robust guidance for the design of AI-integrated learning ecosystems.
